# Specific heat capacity of molten salt-based alumina nanofluid

**DOI:** 10.1186/1556-276X-8-292

**Published:** 2013-06-21

**Authors:** Ming-Chang Lu, Chien-Hsun Huang

**Affiliations:** 1 Department of Mechanical Engineering, National Chiao Tung University, 1001 University Road, Hsinchu 30010, Taiwan

**Keywords:** Nanofluid, Nanolayer, Specific heat capacity (SHC), Molten-salt

## Abstract

There is no consensus on the effect of nanoparticle (NP) addition on the specific heat capacity (SHC) of fluids. In addition, the predictions from the existing model have a large discrepancy from the measured SHCs in nanofluids. We show that the SHC of the molten salt-based alumina nanofluid decreases with reducing particle size and increasing particle concentration. The NP size-dependent SHC is resulted from an augmentation of the nanolayer effect as particle size reduces. A model considering the nanolayer effect which supports the experimental results was proposed.

## Background

While nanofluids, i.e., solvents doped with suspended nanoparticles (NPs), show enhanced thermal conductivities [1-5], the effect of nanoparticle addition on the specific heat capacity (SHC) of the fluids does not provide consistent findings in previous studies [6-12]. For example, Das and co-workers [6-8] found reduced SHCs of nanofluids consisting of silicon dioxide, zinc oxide, and alumina NPs, respectively, dispersed in a mixture of water and ethylene glycol as compared to that of the base fluid. Meanwhile, the SHC of the nanofluid decreases with increasing NP concentration. Zhou and Ni [9] also found a reduced SHC of the water-based alumina nanofluid, and a similar decrease of SHC with increasing particle concentration was observed. In contrast, Zhou et al. [10] found a maximum of 6.25% enhancement of the SHC of the ethylene glycol-based CuO nanofluid. In addition, Shin and Banerjee [11,12] obtained 14.5% and 19% to 24% enhancements of the SHCs in the nanofluids consisting of 1-wt.% SiO_2_ NPs doped in Li_2_CO_3_-K_2_CO_3_ eutectic and chloride eutectic, respectively. Besides, studies [6,10-12] also found a large discrepancy between their experimental results and the predictions from the existing model [13]:

(1) $$c_{p,\mathrm{nf}}=\frac{\phi_{\mathrm{np}}\rho_{\mathrm{np}}c_{p,\mathrm{np}}+\phi_{f}\rho_{f}c_{p,f}}{\phi_{\mathrm{np}}\rho_{\mathrm{np}}+\phi_{f}\rho_{f}},$$

where the subscripts nf, np, and f denote nanofluid, NP, and solvent, respectively, and *c*_p_, *ϕ*, and *ρ* are SHC, volume fraction, and density, respectively. In this work, we investigate SHCs of molten salt-doped with alumina NPs. The material selected is because of the fluid utilized as a heat storage medium in the solar-thermal power plants, and the SHC of it determines energy storage capacity in the power plants. Here, the effect of NP addition on the SHC of the molten salt and the underlying mechanisms were examined. Furthermore, a theoretical model supporting the experimental results was proposed.

## Methods

**Figure 1:**
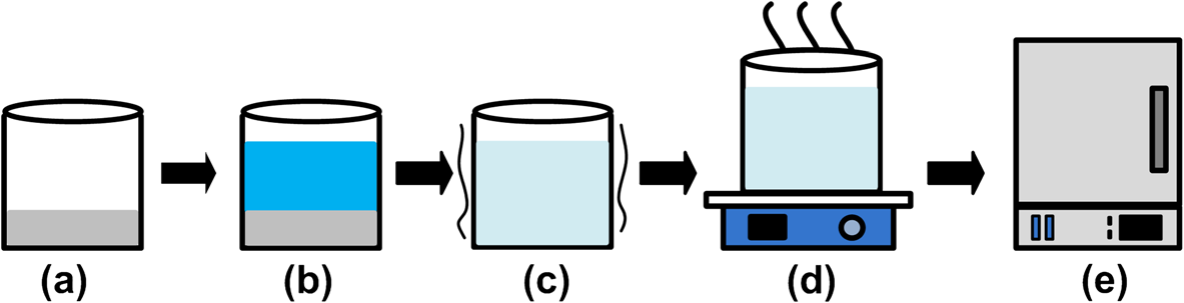
**Nanofluid synthesis.** (**a**) A mixture of molten salt powder and alumina nanoparticles was prepared in a beaker; (**b**) The same weight of DI water was added into the beaker; (**c**) The solution was mixed up in an ultrasonic for 100 min; (**d**) The DI water was evaporated via heating the solution on a hot plate at 90°C for 12 h; (**e**) The solution was melted at 300°C in a high-temperature oven.

The nanofluids were synthesized by introducing various concentrations of the alumina NPs with two nominal sizes of 13 and 90 nm (bought from Sigma-Aldrich, St. Louis, MO, USA) into the molten salt consisting of 60-wt.% NaNO_3_ and 40-wt.% KNO_3_ (i.e., solar salt [14]). The method of nanofluid synthesis is similar to that adopted by Shin and Banerjee [11]. Figure 1 shows the procedure of nanofluid synthesis. First, a mixture of salt (60-wt.% NaNO_3_ and 40-wt.% KNO_3_) and alumina NPs with specified concentration was prepared in a beaker. Second, the same weight of deionized (DI) water was then added into the beaker. Third, the solution was mixed up in an ultrasonic for 100 min. Forth, the DI water was evaporated by heating the solution on a hot plate at 105°C for 12 h. Finally, the well-mixed mixture consisting of the molten salt doped with NPs was melted at 300°C for 40 min in a high-temperature oven. Accordingly, the molten salt-based alumina nanofluid can be obtained.

**Figure 2:**
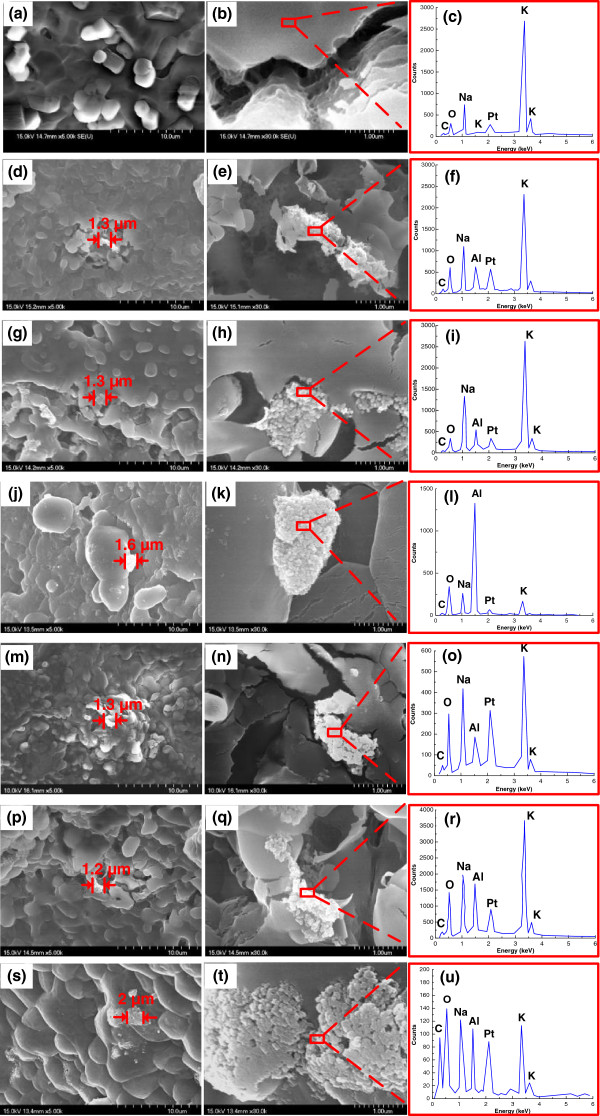
**SEM images and EDS results.** (**a**,**b**) molten salt (×5,000 and × 30,000, respectively); (**d**,**e**) molten salt-based nanofluid containing 13-nm alumina NPs at 0.9 vol.% (×5,000 and × 30,000, respectively); (**g**,**h**) molten salt-based nanofluid containing 13-nm alumina NPs at 2.7 vol.% (×5,000 and × 30,000, respectively); (**j**,**k**) molten salt-based nanofluid containing 13-nm alumina NPs at 4.6 vol.% (×5,000 and × 30,000, respectively); (**m**,**n**) molten salt-based nanofluid containing 90-nm alumina NPs at 0.9 vol.% (×5,000 and × 30,000, respectively); (**p**,**q**) molten salt-based nanofluid containing 90-nm alumina NPs at 2.7 vol.% (×5,000 and × 30,000, respectively); (**s**,**t**) molten salt-based nanofluid containing 90-nm alumina NPs at 4.6 vol.% (×5,000 and × 30,000, respectively), and (**c**,**f**,**i**,**l**,**o**,**r**, and **u**) EDS analysis results at the scanned areas.

The scanning electron microscope (SEM) pictures of the molten salt and nanofluids and corresponding energy dispersive spectrometer (EDS) are shown in Figure 2. Figure 2a,b shows the SEM images for the molten salt under two different magnifications (×5,000 and × 30,000), and Figure 2c is the EDS analysis results at the scanned area outlined in Figure 2b. The EDS results confirm the chemical composition of the molten salt (60-wt.% NaNO_3_ and 40-wt.% KNO_3_). The Pt peak in Figure 2c is from the Pt coating for taking the SEM images while the C peak in Figure 2c is from the carbon paste for SEM sample preparation. Figure 2d,e,g,h,j,k shows the SEM images of the nanofluids containing 13-nm alumina NPs at 0.9, 2.7, and 4.6 vol.%, respectively, under the two different magnifications. Meanwhile, Figure 2f,i,l shows the EDS analysis results at the scanned areas outlined at Figure 2e,h,k. Furthermore, Figure 2m,n,p,q,s,t shows the SEM images of the nanofluids containing 90-nm alumina NPs at 0.9, 2.7, and 4.6 vol.%, respectively, under the two different magnifications. The chemical composition of alumina NPs could be verified by the EDS results shown in Figure 2f,i,l,o,r,u. It is worth noting that the aggregation of NPs was found in the nanofluids when they are in solid state. Meanwhile, the sizes of the clusters formed from the aggregated NPs for the nanofluids in solid state are on the order of 1 μm (see Figure 2d,g,j,m,p,s).

**Figure 3:**
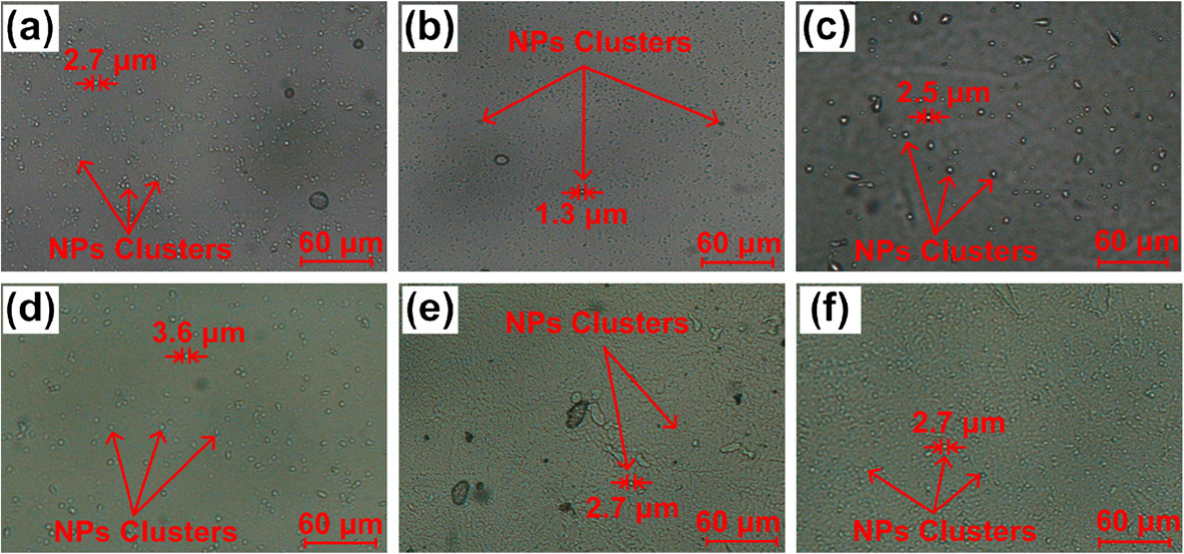
**OM images of nanofluids when in liquid state.** (**a**,**b**,**c**) OM images of the nanofluids containing 13-nm alumina NPs at 0.9, 2.7, and 4.6 vol.%, respectively, and (**d**,**e**,**f**) OM images of the nanofluids containing 90-nm alumina NPs at 0.9, 2.7, and 4.6 vol.%, respectively.

Figure 3 shows the images of the nanofluids in their liquid state. The images were taken from an optical microscope (OM) with a × 600 magnification when heating the nanofluids at 300°C (the melting point of the molten salt is about 222°C). Figure 3a,c shows the OM images of the nanofluids containing 13-nm alumina NPs at 0.9, 2.7, and 4.6 vol.%, respectively. Meanwhile, Figure 3d,f show the OM images of the nanofluids containing 90-nm alumina NPs at 0.9, 2.7, and 4.6 vol.%, respectively. The clusters formed from the aggregated particles can be observed when the nanofluids are in their liquid state whereas the sizes of the clusters are also on the order of 1 μm as can be seen in the figure.

## Results and discussion

**Figure 4:**
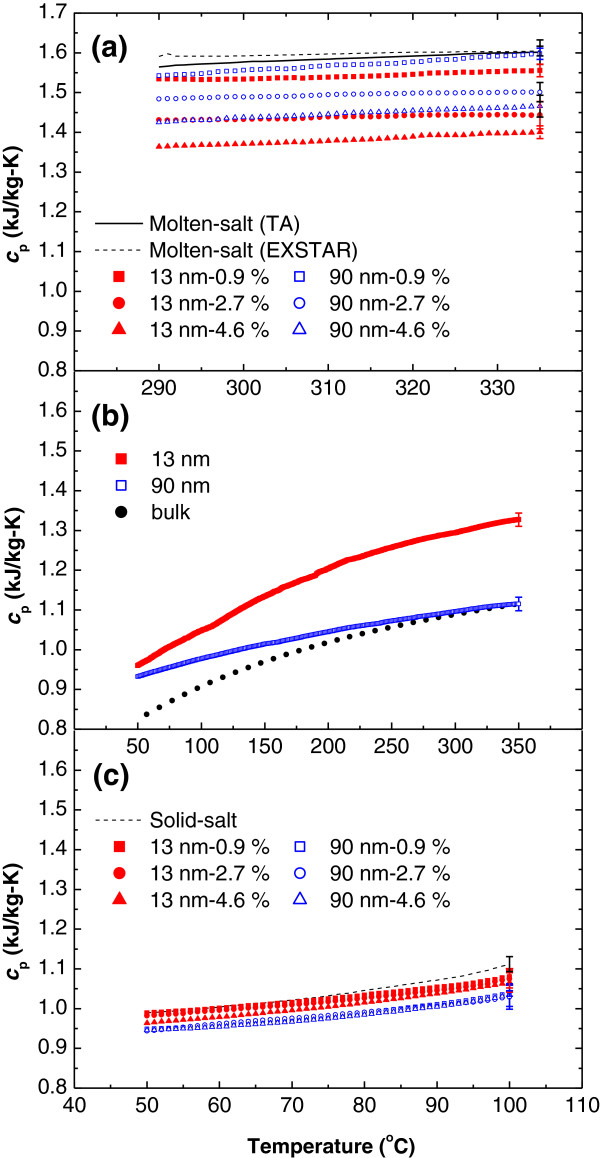
**SHCs of molten salt, nanofluids with alumina NPs, bulk alumina, solid salt, and solid salt doped with alumina NPs.** (**a**) molten-salt (solid and dash lines, measured using Q20 of TA and 7020 of EXSTAR, respectively) and nanofluids having 13-nm alumina NPs at 0.9 (red solid square), 2.7 (red solid circle), and 4.6 vol.% (red solid triangle), respectively, and nanofluids having 90-nm alumina NPs at 0.9 (blue open square), 2.7 (blue open circle), and 4.6 vol.% (blue open triangle), respectively; (**b**) 13-nm alumina NP (red solid square), 90-nm alumina NP (blue open square), and bulk alumina (dark solid circle) [16]; and (**c**) solid salt (dark dash line) and solid salt doped with 13-nm alumina NPs at 0.9 (red solid square), 2.7 (red solid circle), and 4.6 vol.% (red solid triangle), respectively, and 90-nm alumina NPs at 0.9 (blue open square), 2.7 (blue open circle), and 4.6 vol.% (blue open triangle), respectively.

The SHCs of the NPs, molten salt, solid salt doped with NPs, and nanofluids were measured using differential scanning calorimetry (DSC, Model Q20, TA Instrument, New Castle, DE, USA and Model 7020 of EXSTAR, Hitachi High-Tech Science Corporation, Tokyo, Japan). The solid and dash lines in Figure 4a are the SHCs of the molten salt measured using model Q20 of TA and model 7020 of EXSTAR, respectively. In the figure, the SHCs were taken from the average of at least three measurements, and the error bars shown in the figure are the stand errors of these measurements. The SHCs nanofluids having 13-nm and 90-nm alumina NPs at 0.9, 2.7, and 4.6 vol.%, respectively (measured using Q20 of TA) are also shown in Figure 4a. The temperature effect on the SHCs of the molten salt and the nanofluids is not significant as shown in Figure 4a. This is similar to the previous observation for the nitrate salts of NaNO_3_ and KNO_3_, respectively [15]. The 290°C to 335°C temperature-averaged SHCs of the molten salt measured using model Q20 of TA and model 7020 of EXSTAR are similar (1.59 ± 0.031 and 1.60 ± 0.012 kJ/kg-K, respectively). These values are similar to the value (1.55 kJ/kg-K) reported from Coastal Chemical for the molten salt [14]. These also validate our DSC measurements.

Figure 4b shows the SHCs of the 13-nm and 90-nm alumina NPs and bulk alumina at various temperatures. The SHCs of NPs were measured using model 7020 of EXSTAR while the values of the SHCs of the bulk alumina were taken from Ginnings and Furukawa [16]. The SHCs of NPs and bulk alumina increases as temperature increases. Meanwhile, the SHC increases as particle size reduces and the SHC of 90 nm is approaching that of bulk alumina at high temperature. The size-dependent SHC of NPs has also been observed from previous studies [17,18]. This increment of SHC with reducing particle size could be explained by the Debye model of heat capacity of solids, wherein the heat capacity increases as the Debye temperature reduces [18]. The Debye temperature decreases with reducing particle size [17], resulting in an increased SHC.

Figure 4c shows the SHCs of solid salt and solid salt doped with 13-nm and 90-nm alumina NPs at 0.9, 2.7, and 4.6 vol.%, respectively (measured using model 7020 of EXSTAR). The effect of NP concentration on the SHC of the solid salt doped with NPs is not significant whereas the SHC decreases with increasing NP size. The NP-size-dependent SHC might be due to the fact that the larger NPs have a smaller SHC (see Figure 4b). Nevertheless, the effect of NP addition on the SHC of the nanofluid is pronounced (see Figure 4a).

**Figure 5:**
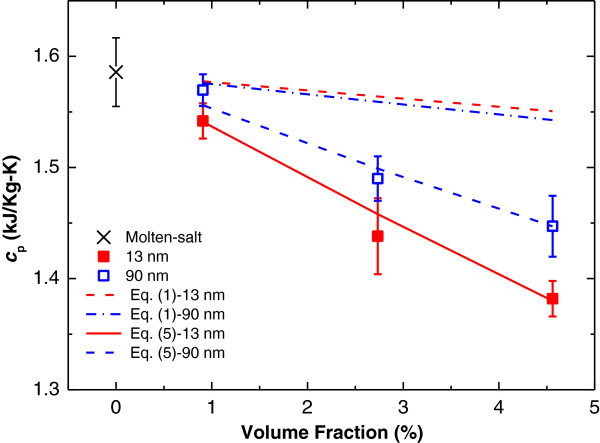
**Effects of NP size and concentration on the SHC of the nanofluid.** The cross mark at 0 vol.% is the SHC of the molten salt without doping with NPs (measured using model Q20 of TA). The red dash and blue dash-dot lines show the model prediction using Equation 1 for 13- and 90-nm alumina NPs at various volume fractions. The red solid squares and blue open squares are the experimental results of the SHCs of the nanofluids having 13- and 90-nm alumina NPs at 0.9, 2.7, and 4.6 vol.%, respectively. The red solid line and blue dash line are the model predictions considering the nanolayer effect on the SHC of the nanofluid (Equation 5).

The effects of size and concentration of the NPs on the SHCs of the nanofluids are illustrated in Figure 5. The temperature-averaged SHCs of nanofluids between 290°C and 335°C were taken to evaluate the effectiveness on the energy storage of the nanofluids in the temperature range. The cross mark data at 0 vol.% in Figure 5 is the SHC of the molten salt without doping with NPs (measured using model Q20 of TA). The red solid squares and blue open squares are the experimental results of the temperature-averaged SHCs of the nanofluids having 13-nm and 90-nm alumina NPs at 0.9, 2.7, and 4.6 vol.%, respectively. A reduced SHC of the nanofluid as compared to that of the base fluid is observed, and the SHC of the nanofluid decreases with increasing NP concentration, which is similar to previous studies [6-10]. Furthermore, the SHC of the nanofluids is particle-size-dependent. The SHC decreases with reducing particle size, in contrast to the trend observed in the solid salt doped with NPs (see Figure 4c). The particle-size-dependent SHC in nanofluids had never been reported before and could not be explained by the size-dependent SHCs of alumina NPs since smaller NP has a larger SHC (see Figure 4b).

The theoretical prediction using Equation 1 is also shown in Figure 5, where the values of *c*_p,np_ are obtained from the temperature-averaged (290°C to 335°C) SHCs of the 13- and 90-nm alumina NPs shown in the Figure 4b (i.e., 1.30 and 1.10 kJ/kg-K, respectively). The red dash line and blue dash-dot line in Figure 5 are the theoretical predictions of Equation 1 for the nanofluids having 13- and 90-nm alumina NPs, respectively (where *c*_p,13nm_, *c*_p,90nm_, and *c*_p,f_ are 1.30, 1.10, and 1.59 kJ/kg-K, respectively whereas *ρ*_np_ and *ρ*_f_ are 3,970 and 1794 kg/m^3^, respectively). It is noted that the alumina NP density was taken from the value of the bulk alumina as an approximation. The existing model (Equation 1) predicts a slight decrease trend of the SHC of the nanofluid with increasing particle concentration since the SHCs of NPs are smaller than that of molten salt. This slight decrease tread is similar to that observed for the solid salt doped with NPs (see Figure 4c). Furthermore, the model (Equation 1) shows that the SHCs of nanofluids decrease with increasing particle size because smaller particles have larger SHC, which is in contrast to the experimental results for the nanofluid. In addition, the experimental results have a large difference from the model prediction of Equation 1, which has also been observed in previous studies [6,9-12]. This indicates that there might be other mechanisms responsible for the large discrepancy.

The proposed mechanisms for the thermal conductivity enhancement are the following: (1) Brownian motion [19,20]. It is argued that Brownian motion of NPs in the solvent could result in a microconvection effect that enhances heat transfer of the fluid; (2) Colloidal effect [21-23]. It says that heat transfer in nanofluids can be enhanced by the aggregation of NPs into clusters; (3) Nanolayer effect [24-26]. The solid-like nanolayer formed on the surface of the nanoparticle could enhance the thermal conductivity of the fluid [14]. In light of these studies, we believe that some of these mechanisms might affect the SHC of nanofluid as well.

Particle aggregation was observed when both the solid salt and the molten salt were doped with NPs as shown in Figures 2 and 3. The sizes of the clusters formed from the aggregated NPs are both on the order of 1 μm in the solid salt and molten salt (see Figures 2 and 3). However, the SHC of the solid salt doped with NPs is close to that of solid salt alone whereas the SHC of the molten salt doped with NPs is apparently different from that of molten salt. Furthermore, the NP size effect shows reverse trends in these two cases: the SHC of solid salt increases as NP size reduces (see Figure 4c) whereas the SHC of molten salt doped with NPs decreases as NP size reduces (see Figure 4a). This indicates that the observed large discrepancy between the SHCs of nanofluid and molten salt does not result from the particle aggregation effect. In addition, Ishida and Rimdusit [27] have also shown that the SHC is a structure-insensitive property, provided that formation of different degrees of network do not affect the SHC of the composite. Furthermore, since SHC is not a transport property, the microconvection effect caused by Brownian motion of NPs should not play a significant role in the SHC of the nanofluid. Thus, we conjectured that the nanolayer effect might be the only important factor among these three mechanisms affecting the SHC of the nanofluid. Accordingly, a theoretical model considering the nanolayer effect on the SHC was proposed. Since the solid-like nanolayer formed on the surface of NP is at a thermodynamic state between solid salt and molten salt [26], the value of the SHC of the nanolayer should lay between those of the solid salt (1.04 kJ/kg-K) and the molten salt (1.59 kJ/kg-K). In other words, the nanolayer has a lower SHC than that of the molten salt. Further, the thermal properties of the nanolayer (e.g., thermal conductivity and SHC) could vary with different combinations of NPs and base fluids, since the structure of the nanolayer is dependent on the interaction of molecules [28]. In addition, Lin et al. [25] also found that the nanolayer structure is size-dependent, resulting in a size-dependent thermal conductivity.

As a result, the SHC of the nanolayer is dependent on the size of the NP and the combinations of the NPs and base fluids. To the best of our knowledge, there is no experimental and theoretical data available for the SHC of the nanolayer for the molten salt-based alumina nanofluid. Thus, in this proposed model, the SHC of the nanolayer (*c*_p,layer_) for a given NP size is first obtained from the experimental result of the SHC of the nanofluid at a certain particle concentration (i.e., *c*_p,m_):

(2) $$c_{p,m}=\frac{c_{p,\text{layer}}W_{\text{layer}}+c_{p,\text{np}}W_{\text{np}}+c_{p,f}\left(W_{f}-W_{\text{layer}}\right)}{W_{\text{nf}}},$$

where the subscript layer is denoted as nanolayer; *W* is weight; and *W*_nf_ = *W*_np_ + *W*_f_. In the model, it is assumed that the measured SHC of the nanofluid (*c*_p,m_) is a result of the superposition of the SHCs of the nanolayer (*c*_p,layer_), the NP (*c*_p,np_), and the solvent (*c*_p,f_) as in contrast to the existing model (Equation 1). Thus, the SHC of the nanolayer (*c*_p,layer_) for the given NP size could be obtained from Equation 2:

(3) $$c_{p,\text{layer}}=\frac{c_{p,m}W_{\mathrm{nf}}-c_{p,\mathrm{np}}W_{\mathrm{np}}-c_{p,f}\left(W_{f}-W_{\text{layer}}\right)}{W_{\mathrm{layer}}},$$

Once the SHC of the nanolayer was known, the SHC of the nanofluid (*c*_p,nf_) at any NP concentration (having a mass fraction *α’* = *W*_np_*’*/*W*_nf_*’*) for the given NP size could be obtained as follows:

(4) cp,nf=cp,npWnp'+cp,layerWlayer'+cp,fWf−Wlayer'Wnf',

where *W*_np_*’*, *W*_layer_*’*, and *W*_nf_*’* are the weights of NP, nanolayer, and nanofluid at such NP concentration, respectively. Meanwhile, the weight of solvent (*W*_f_) is kept as a constant for various particle concentrations. Substituting *c*_p,layer_ from Equation 3 into Equation 4, one can obtain the SHC of the nanofluid for the given NP size at such NP mass fraction (α*’* = *W*_np_*’*/*W*_nf_*’*) as follows:

(5) $$c_{p,\mathrm{nf}}=\frac{c_{p,f}\left(\alpha -\alpha '\right)+c_{p,}{}_{m}\alpha '}{\alpha },$$

where *α* ( = *W*_np_/*W*_nf_) is the NP mass fraction in determining SHC of the nanolayer in Equations 2 and 3 and the SHC of the solvent (*c*_p,f_) was obtained from the DSC measurements (*c*_p,f_ = 1.59 kJ/kg-K). It is worth noting that the SHCs of the NPs and nanolayer are not required for the theoretical prediction using Equation 5, of which the effects on the SHC of the nanofluid are implicitly included in the term *c*_p,m_ in Equation 5. For the condition *α* = *α’*, one recovers *c*_p,nf_ = *c*_p,m_ as expected. The relation between volume fraction and mass fraction is as follows:

(6) $$\phi =\alpha^{2}\;\left(1-\frac{\rho_{f}}{\rho_{\mathrm{np}}}\right)+\alpha \;\left(\frac{\rho_{f}}{\rho_{\mathrm{np}}}\right)\;,$$

where *ρ*_f_ and *ρ*_np_ are solvent density and NP density, respectively.

Using Equation 5, one can obtain the SHC of the nanofluid (*c*_p,nf_) at any mass fraction (*α’*) from the measured SHC of the nanofluid (*c*_p,m_) at a certain mass fraction (*α*) for a given NP size. The predictions using Equation 5 for the SHCs of the nanofluids at various concentrations having 13-nm alumina NPs (red solid line) and 90-nm alumina NPs (blue dash line) based on the measured SHCs at 4.6 vol.%, along with the experimental results, are also shown in Figure 5. As Figure 5 shows, the predictions from the proposed model agree well with the experimental results.

The large difference between the predictions of Equations 5 and 1 is from the result of the nanolayer effect on the SHC. This could be better understood by looking at the third term in the numerator of Equation 4. Since the weight of nanolayers (*W*_layer_*’*) increases as particle concentration increases, it results in a further reduced SHC, provided that the nanolayer has a lower SHC than that of molten salt. Furthermore, the increase of SHC with increasing particle size is also a result of the nanolayer effect. For a given NP concentration, the nanolayer effect increases as particle size reduces since the number of particle increases with reducing particle size. Thus, one observes a decreased SHC as particle size reduces, and particle concentration increases because of the augmentation of the nanolayer effect.

## Conclusions

In conclusion, we have explored the SHC of the molten salt-based alumina nanofluid. The NP size-dependent SHC in the nanofluids had never been reported before and cannot be explained by the current existing model. We found that the reduction of the SHC of nanofluid when NP size reduces is due to the nanolayer effect, since the nanolayer contribution increases as particle size reduces for a given volume fraction. A theoretical model taking into account the nanolayer effect on the SHC of nanofluid was proposed. The model supports the experimental results in contrast to the existing model. The findings from this study are advantageous for the evaluation of the application of nanofluids in thermal storage for solar-thermal power plants.

## Abbreviations

NP: Nanoparticle; SHC: Specific heat capacity; DI: Deionized; SEM: Scanning electron microscope; EDS: Energy dispersive spectrometer; OM: Optical microscope; DSC: Differential scanning calorimetry.

## Competing interests

The authors declare that they have no competing interests.

## Authors’ contributions

The manuscript was written through contributions of all authors. All authors have given approval to the final version of the manuscript.
